# Online behavioural interventions for children and young people with eczema: a quantitative evaluation

**DOI:** 10.3399/BJGP.2023.0411

**Published:** 2024-05-31

**Authors:** Kate Greenwell, Taeko Becque, Katy Sivyer, Mary Steele, James Denison-Day, Laura Howells, Matthew J Ridd, Amanda Roberts, Sandra Lawton, Sinéad M Langan, Julie Hooper, Sylvia Wilczynska, Gareth Griffiths, Tracey H Sach, Paul Little, Hywel C Williams, Kim S Thomas, Lucy Yardley, Ingrid Muller, Miriam Santer, Beth Stuart

**Affiliations:** Primary Care Research Centre, Primary Care, Population Sciences and Medical Education Unit, Faculty of Medicine; Centre for Clinical and Community Applications of Health Psychology, Faculty of Environmental and Life Sciences, University of Southampton, Southampton.; Primary Care Research Centre, Primary Care, Population Sciences and Medical Education Unit, Faculty of Medicine, University of Southampton, Southampton.; Centre for Clinical and Community Applications of Health Psychology, Faculty of Environmental and Life Sciences, University of Southampton, Southampton.; Primary Care Research Centre, Primary Care, Population Sciences and Medical Education Unit, Faculty of Medicine, University of Southampton, Southampton.; Centre for Clinical and Community Applications of Health Psychology, Faculty of Environmental and Life Sciences, University of Southampton, Southampton.; Centre of Evidence Based Dermatology, Lifespan and Population Health, School of Medicine, University of Nottingham, Nottingham.; Population Health Sciences, University of Bristol, Bristol.; Centre of Evidence Based Dermatology, Lifespan and Population Health, School of Medicine, University of Nottingham, Nottingham.; Queen’s Nurse, Department of Dermatology, Rotherham NHS Foundation Trust, Rotherham.; Faculty of Epidemiology and Population Health, London School of Hygiene and Tropical Medicine, London.; Primary Care Research Centre, Primary Care, Population Sciences and Medical Education Unit, Faculty of Medicine, University of Southampton, Southampton.; King’s Clinical Trials Unit, King’s College London.; Southampton Clinical Trials Unit, University of Southampton, Southampton.; Primary Care Research Centre, Primary Care, Population Sciences and Medical Education Unit, Faculty of Medicine, University of Southampton, Southampton.; Primary Care Research Centre, Primary Care, Population Sciences and Medical Education Unit, Faculty of Medicine, University of Southampton, Southampton.; Centre of Evidence Based Dermatology, Lifespan and Population Health, School of Medicine, University of Nottingham, Nottingham.; Centre of Evidence Based Dermatology, Lifespan and Population Health, School of Medicine, University of Nottingham, Nottingham.; Centre for Clinical and Community Applications of Health Psychology, Faculty of Environmental and Life Sciences, University of Southampton, Southampton; School of Psychological Science, University of Bristol, Bristol.; Primary Care Research Centre, Primary Care, Population Sciences and Medical Education Unit, Faculty of Medicine, University of Southampton, Southampton.; Primary Care Research Centre, Primary Care, Population Sciences and Medical Education Unit, Faculty of Medicine, University of Southampton, Southampton.; Centre for Evaluation and Methods, Wolfson Institute of Population Health, Faculty of Medicine and Dentistry, Queen Mary University of London.

**Keywords:** eczema, internet, patient education, primary care, self-management

## Abstract

**Background:**

Two online behavioural interventions (one website for parents/carers of children with eczema; and one for young people with eczema) have been shown in randomised controlled trials to facilitate a sustained improvement in eczema severity.

**Aim:**

To describe intervention use and examine potential mediators of intervention outcomes and contextual factors that may influence intervention delivery and outcomes.

**Design and setting:**

Quantitative process evaluation in UK primary care.

**Method:**

Parents/carers and young people were recruited through primary care. Intervention use was recorded and summarised descriptively. Logistic regression explored sociodemographic and other factors associated with intervention engagement. Mediation analysis investigated whether patient enablement (ability to understand and cope with health issues), treatment use, and barriers to adherence were mediators of intervention effect. Subgroup analysis compared intervention effects among pre-specified participant subsets.

**Results:**

A total of 340 parents/carers and 337 young people were recruited. Most parents/carers (87%, *n* = 148/171) and young people (91%, *n* = 153/168) in the intervention group viewed the core introduction by 24 weeks. At 24 weeks, users had spent approximately 20 minutes on average on the interventions. Among parents/carers, greater intervention engagement was associated with higher education levels, uncertainty about carrying out treatments, and doubts about treatment efficacy at baseline. Among young people, higher intervention use was associated with higher baseline eczema severity. Patient enablement (the ability to understand and cope with health issues) accounted for approximately 30% of the intervention effect among parents/carers and 50% among young people.

**Conclusion:**

This study demonstrated that positive intervention outcomes depended on a modest time commitment from users. This provides further support that the wider implementation of Eczema Care Online is justified.

## Introduction

Eczema (also known as atopic eczema/dermatitis) is a common long-term skin condition characterised by itchy, dry, and inflamed skin. It affects around 20% of children in the UK and often persists into adulthood.^1^ Eczema can have substantial impact on quality of life for those affected and their families.^2^^,^^3^ First-line eczema treatment includes emollients and topical corticosteroids alongside the avoidance of irritants/triggers (such as soap).^4^ Topical treatments are often underused because of uncertainty and hesitancy, and irritants and triggers are often not well understood.^5^^,^^6^ While there is evidence to suggest that intensive group education delivered by a multidisciplinary team can improve outcomes in eczema,^7^ less is known about the effectiveness of online behavioural interventions.^8^^,^^9^

The authors developed two online behavioural interventions (called Eczema Care Online): one for parents/carers of children with eczema; and one for young people with eczema.^10^^,^^11^ These interventions were evaluated in two randomised controlled trials (RCTs), which demonstrated that the interventions provided a useful, sustained improvement in the eczema severity symptoms for up to 52 weeks in both children and young people, when offered in addition to usual care. The mean difference in Patient-Oriented Eczema Measure (POEM) score was −1.5 (95% confidence interval [CI] = −2.5 to −0.6; *P* = 0.002) in the parents/carers trial, and −1.9 (95% CI = −3.0 to −0.8; *P*<0.001) in the young people trial.^12^ These RCTs also explored the impact of the interventions on two hypothesised behavioural mechanisms: emollient use and topical corticosteroid/topical calcineurin inhibitor use; as well as two psychological mechanisms: patient enablement (the self-perceived ability to understand and cope with health issues) and perceived barriers to treatment. There were no significant differences between groups found for either RCT in self-reported treatment use at 24 weeks or perceived barriers to treatments at 24 weeks.^12^ However, improvements in patient enablement were found in the intervention groups in both trials: adjusted mean difference at 24 weeks was −0.7 (95% CI = −1.0 to −0.4) for parents/carers and −0.9 (95% CI = −1.3 to −0.6) for young people.^12^

**Table:** How this fits in

Two randomised controlled trials demonstrated that online behavioural interventions (one for parents/carers of children with eczema and another for young people with eczema) provided useful, sustained improvements in eczema severity. There is a need to develop an in-depth understanding of how such interventions work and the contextual factors influencing their delivery. Users spent approximately 20 minutes on the interventions on average, demonstrating that positive outcomes could be achieved with relatively little time commitment. The intervention effect on eczema severity was mediated principally by improvement to an individual’s ability to understand and cope with health issues.

A qualitative process evaluation provided further support for the role of patient enablement.^13^ Specifically, parents/carers and young people reported that the interventions supported them to feel confident in managing eczema and discussing treatments with healthcare professionals, to normalise and accept eczema, and (for parents) to involve their child in eczema management.

In line with the Medical Research Council guidelines for developing and evaluating complex interventions,^14^^,^^15^ the current study involved a quantitative process evaluation to further explore how the interventions worked and for whom. These guidelines recommend exploring:

1. implementation: the extent to which the intervention is used as intended;
2. mechanisms: the processes by which an intervention leads to changes in the intended outcome; and
3. context: aspects of the target population or setting that may have influenced intervention delivery or outcomes.

For both parents/carers and young people groups, this study therefore aimed to:

1. describe intervention use for those allocated to the intervention group (implementation);
2. examine whether any of patient enablement, treatment use, or perceived barriers to treatment mediated the relationship between the intervention and the outcome of eczema severity (mechanisms); and
3. examine whether user demographics (such as age, sex, education, and level of deprivation), baseline levels of treatment use, and baseline perceived barriers to treatment are associated with use of key intervention content and intervention outcome (eczema severity) (context).

## Method

### Design

This study involved a quantitative process evaluation embedded within two RCTs (details of which are reported elsewhere).^12^^,^^16^ Trial participants were randomised into either an intervention group who were given access to the relevant online intervention in addition to usual care, or into a usual care group who received usual care and were recommended a standard informational website (https://eczema.org), and given access to the intervention on study completion.

### Intervention

The two digital interventions were developed using evidence-, theory-, and person-based approaches.^17^^,^^18^ The aim of both interventions was to reduce eczema severity via several behavioural mechanisms applying to children and young people: increased use of emollients; improved use of topical corticosteroids or topical calcineurin inhibitors; improved management of irritants and triggers; reduced scratching; and improved emotional management. The first two mechanisms were identified as core behaviours likely to have the greatest effect on eczema severity.^10^^,^^11^ Intervention development and the content and design features are summarised in Box 1 and described in detail elsewhere.^10^^,^^11^ See Supplementary Figures S1 and S2 for details of logic models of hypothesised intervention mechanisms.

**Box 1.** Summary of key intervention content and design features

**Intervention content**
Intervention content encouraged users to engage in five target behaviours: use of emollient; use of topical corticosteroids or topical calcineurin inhibitors during a flare-up; management of irritants and triggers; reduced scratching (children and young people); and emotional management (children and young people).
**Key design features**
Websites accessible via a mobile device At the beginning of the intervention, users first progressed through a brief (9 pages) introductory section containing the key content necessary for facilitating behaviour change Two core modules about topical treatments: emollients and topical corticosteroids or topical calcineurin inhibitors Optional modules (14 in young people intervention and 16 in parent/carer intervention) Videos (four in young people intervention and five in parent/child intervention; lasting approximately 2 minutes) briefly summarising key behavioural messages A ‘2-week challenge’ that supported people to get into a routine of applying emollients consistently A brief eczema assessment that provided tailored advice on which treatment modules (emollients or flare control creams) would be most helpful Quotes from other parents/carers and young people with eczema that share their experiences of eczema and management advice Monthly automated email or SMS with additional information and advice for 6 months
The two tested websites have since been combined into one (https://www.eczemacareonline.org.uk).

### Recruitment

Trial participants were recruited from GP practices in England. Participants were invited through a search of electronic health records and postal invitation. For children younger than 16 years, the invitation was sent to their parent or carer. Participants were eligible if they were a parent/carer of a child aged 0–12 years with eczema or a young person aged 13–25 years with eczema, who had obtained a relevant prescription in the previous 12 months. Eczema severity was assessed online at screening and those with very mild or inactive eczema (POEM score ≤5)^19^^,^^20^ were excluded. Parents/carers and young people aged ≥16 years consented online. Parents/carers of young people aged 13–15 years provided online consent for their child, in addition to assent being sought from the child.

### Data collection

Measures and their timepoints are summarised in Table 1. Intervention use data were automatically collected using LifeGuide software (https://www.lifeguideonline.org), which was used to create and host the intervention. When exploring engagement with digital interventions, it has been argued that the focus should be on ‘effective engagement’, defined as the minimal level of engagement necessary for achieving the intended outcomes of the intervention, rather than broad engagement measures, such as number of logins or time spent on the intervention.^21^ The effective engagement threshold may involve viewing certain intervention content that is judged to be most likely to lead to behaviour change.^22^ Following the AMUsED (Analyzing and Measuring Usage and Engagement Data) framework^22^ for analysing and measuring engagement data in digital interventions, the authors chose two patterns of intervention use to explore as potential effective engagement thresholds. The first was viewing the core introductory content that contains the key content the authors deemed necessary for behaviour change (minimum engagement threshold). The second was viewing the core introductory content and at least one optional module (higher engagement threshold).

**Table 1.** Quantitative measures and timepoints

**Variable**	**Measure**	**Timepoint**
**Implementation**		
Website use (intervention group only)	Objective data automatically recorded by intervention software (number, time and duration of logins, pages visited, and time spent on each page)	All use across 52-week period^a^

**Mechanisms and contextual factors**		
Patient enablement (mechanisms) Demographics (context)	The Patient Enablement Instrument (PEI) Self-reported age, sex, education (parents/carers), and Index of Multiple Deprivation quintile (derived from postcode)	Baseline, 24 weeks Baseline
Weekly emollient use (mechanisms and context)	2-item self-report questionnaire (number of days per week and number of times per day)	Baseline, 24 weeks
Weekly topical corticosteroid use (mechanisms and context)	1-item self-report questionnaire	Baseline, 24 weeks
Weekly topical calcineurin inhibitor use (mechanisms and context)	1-item self-report questionnaire	Baseline, 24 weeks
Perceived barriers to treatment (mechanisms and context)	12-item Problematic Experiences of Therapy Scale (PETS) with subscales: symptoms too severe, uncertainty how to carry out treatment, doubts about treatment efficacy, and practical problems	Baseline, 24 weeks

**Intervention (primary) outcome**		
Eczema severity	7-item Patient-Oriented Eczema Measure (POEM)	Baseline, 24 weeks

a *As recruitment start times were staggered, this will be a different 52-week period for each participant.*

Mediators and outcomes were measured at 24 weeks, as this was the primary outcome timepoint in the RCT. The modified Patient Enablement Instrument (PEI)^23^^,^^24^ was tailored to be eczema- and parent/young person-specific, and the question was amended so that responders indicated how they felt ‘as a result of the eczema care and support you have received in the past 6 months’.

### Data analysis

Intervention use at 24 weeks (primary outcome point) and 52 weeks (entire study period) was summarised descriptively. Eczema severity (POEM) was analysed at 24 weeks, to enable mediation and adherence analyses to be carried out, as these cannot be implemented using repeated measures models as were used in the main RCT.^12^

**Figure 1.**
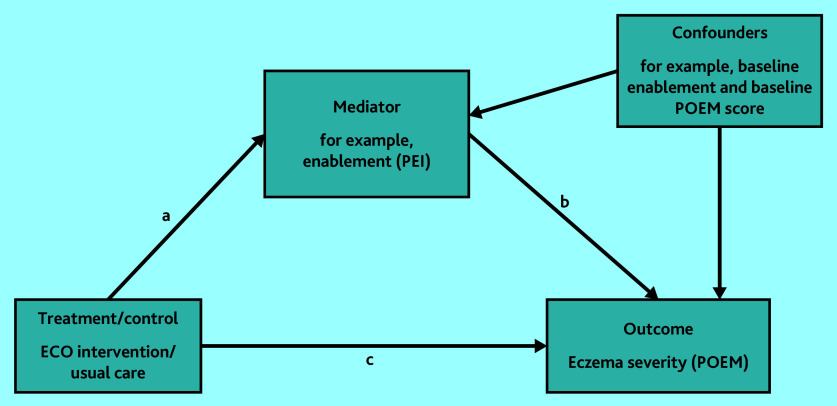
Mediation model estimating direct effect (c), indirect effect (ab), and total effect (ab+c). ECO = Eczema Care Online. PEI = Patient Enablement Instrument. POEM = Patient-Oriented Eczema Measure.

Mediation analysis determined whether patient enablement, treatment use, or barriers to adherence mediated the effect of the intervention on eczema severity. A structural equation model was fitted with the PEI (patient enablement) at 24 weeks as a mediator and POEM score at 24 weeks as the outcome (Figure 1). Because of potential confounding between the mediator and outcome, baseline POEM score and baseline PEI score were also adjusted for in the mediation model (Figure 1). This analysis was repeated using total treatment use and total Problematic Experiences of Therapy Scale (PETS)^25^ score as mediators, adjusting for baseline POEM and baseline treatment use/baseline PETS scores, respectively. Total treatment use was calculated by combining weekly emollient use (total number of times per week), weekly topical corticosteroid use, and weekly topical calcineurin inhibitor use.

Subgroup analysis explored whether the intervention effect was different among people with pre-specified categories of baseline variables: sex, age group, deprivation, baseline eczema severity, emollient use (days per week), and barriers to adherence. The mean difference in POEM score at 24 weeks in each subgroup was reported, along with the interaction term (difference in treatment effect between subgroups) adjusted for baseline POEM score, recruitment region, ethnicity, previous belief in intervention, use of other eczema websites, and highest parental qualification (parent/carer group only). Associations between high intervention use and various demographic and baseline characteristics, including age, sex, education, deprivation, baseline severity, baseline treatment use, and baseline barriers to adherence, were explored using logistic regression.

As the intervention effect at 24 weeks was similar in both the parent/carer and young people trials, sensitivity analyses pooling these participants were also carried out. All analyses were undertaken on complete cases using Stata (version 17), with statistical significance taken as *P*<0.05.

## Results

### Participants

A total of 314/340 (92%) parents/carers and 304/337 (90%) young people completed 24-week POEM (primary timepoint). Overall, 171 parents/carers and 168 young people were allocated to the intervention group (see Supplementary Table S1 for details of baseline participant characteristics).

### Implementation

Table 2 presents summaries of general intervention use for each intervention group across 24 and 52 weeks (see Supplementary Table S2 for details of key intervention component use). Most intervention participants viewed the core introductory content (containing the key content deemed necessary for behaviour change) by 24 weeks (87%, *n* = 148/171 in the parent/carer trial and 91%, *n* = 153/168 in the young people trial) and by 52 weeks (88%, *n* = 151/171 in the parent/carer trial and 93%, *n* = 156/168 in the young people trial). About half of participants viewed the core introductory content and at least one optional module (high users) by 24 weeks (58%, *n* = 99/171 in the parent/carer trial and 57%, *n* = 96/168 in the young people trial) and by 52 weeks (61%, *n* = 104/171 in the parent/carer trial and 59%, *n* = 99/168 in the young people trial).

**Table 2.** General website use across 24 and 52 weeks for both intervention groups

**Variable**	**Parents/carers (*n* = 171)**		**Young people (*n* = 168)**	

	**24 weeks**	**52 weeks**	**24 weeks**	**52 weeks**
**Intervention visits per participant^a^**				
Median (IQR)	3 (3)	5 (7)	3 (4)	4 (7)
Range	0–10	1–17	1–17	1–25

**Duration of intervention use (between first and last use), days^a^**				
Median (IQR)	N/A	252 (201)	N/A	195 (167)
Range	N/A	1–364	N/A	10–364

**Total time spent on intervention, minutes^a^**				
Median (IQR)	20 (36)	27 (41)	18 (32)	21 (36)
Range	2–154	2–157	3–201	3–208

**Modules started per participant^b^**				
Median (IQR)	3 (4)	4 (5)	3 (4)	3 (4)
Range	0–15	0–15	0–17	0–17

**Modules finished per participant^b^**				
Median (IQR)	2 (2)	2 (3)	2 (2)	2 (2)
Range	0–10	0–10	0–16	0–16

a *Excluding visits to complete research questionnaires and users who visited only once.*

b *Total modules for parents/carers is 19; total modules for young people is 17. IQR = interquartile range. N/A = not applicable.*

### Mechanisms

For parents/carers, patient enablement had a statistically significant mediating effect of the intervention on the child’s POEM score (−0.6, 95% CI = −1.0 to −0.2). As a proportion of the total effect, this corresponds to about 30% of the intervention effect on the POEM score at 24 weeks (mediating effect/total effect = −0.6/–1.9) (Table 3). For young people, patient enablement also had a statistically significant mediating effect (−1.2, 95% CI = −1.9 to −0.5). This corresponds to about 50% of the intervention effect on the POEM score at 24 weeks (mediating effect/total effect = −1.2/–2.4). There was no evidence of a mediating effect of total treatment use or perceived barriers to treatment in either trial.

**Table 3.** Mediation models for POEM score at 24 weeks

**Mediator**	**Effect**	**Unadjusted effect (95% CI)**	**Adjusted effect^a^ (95% CI)**
**Parent/carer trial**			

PEI at 24 weeks	Indirect (mediating) effect	−0.7 (−1.1 to −0.2)	**–0.6 (−1.0 to −0.2)**
	Direct effect	−1.3 (−2.8 to 0.2)	−1.3 (−2.6 to −0.1)
	Total effect	−2.0 (−3.4 to −0.5)	−1.9 (−3.2 to −0.7)

Total treatment use at 24 weeks^b^	Indirect (mediating) effect	−0.2 (−0.9 to 0.5)	−0.01 (−0.6 to 0.6)
	Direct effect	−2.0 (−3.5 to −0.5)	−1.5 (−2.9 to −0.1)
	Total effect	−2.2 (−3.8 to −0.6)	−1.5 (−3.0 to 0.003)

PETS score at 24 weeks	Indirect (mediating) effect	0.1 (−0.3 to 0.5)	0.1 (−0.1 to 0.4)
	Direct effect	−1.8 (−3.2 to 0.4)	−1.9 (−3.1 to −0.7)
	Total effect	−1.7 (−3.1 to 0.2)	−1.8 (−3.0 to −0.5)

**Young people trial**			

PEI at 24 weeks	Indirect (mediating) effect	−1.4 (−2.1 to −0.6)	**–1.2 (−1.9 to −0.5)**
	Direct effect	−1.2 (−2.9 to 0.5)	−1.2 (−2.8 to 0.4)
	Total effect	−2.6 (−4.2 to −0.9)	−2.4 (−4.0 to −0.9)

Total treatment use at 24 weeks^b^	Indirect (mediating) effect	−0.01 (−0.5 to 0.5)	−0.2 (−0.7 to 0.3)
		
	Direct effect	−2.0 (−3.7 to −0.4)	−1.7 (−3.3 to −0.2)
	Total effect	−2.0 (−3.7 to −0.3)	−1.9 (−3.5 to −0.3)

PETS score at 24 weeks	Indirect (mediating) effect	−0.1 (−0.5 to 0.3)	−0.1 (−0.4 to 0.2)
	Direct effect	−2.0 (−3.6 to −0.5)	−1.7 (−3.3 to −0.2)
	Total effect	−2.2 (−3.8 to −0.5)	−1.8 (−3.4 to −0.2)

a *Adjusted for baseline POEM score and baseline value of the potential mediator (PEI, total treatment use, or PETS).*

b *Total treatment use is weekly combined emollient, topical corticosteroid, and topical calcineurin inhibitor use. PEI = Patient Enablement Instrument. PETS = Problematic Experiences of Therapy Scale. POEM = Patient-Oriented Eczema Measure. Bold numbers indicate statistical significance.*

### Context

Most subgroup effects were not statistically significant, except for parents/carers with children with severe eczema at baseline, who had a significantly larger treatment effect than those with children with mild eczema at baseline (−4.0 versus 0.8, adjusted interaction term −4.0; 95% CI = −7.7 to −0.2) (see Supplementary Tables S3–S5 for details). This could be because of a floor effect among those with mild eczema.

In the parent/carer trial, having a degree, having uncertainty about how to carry out treatment, and having doubts about treatment efficacy were significantly associated with meeting the higher intervention engagement threshold (Table 4). In the young people trial, higher baseline POEM score (reflecting worse eczema severity) was significantly associated with meeting the higher intervention engagement threshold.

**Table 4.** Predictors of high intervention use

**Variable**	**High user — no**	**High user — yes**	**Odds ratio (95% CI)**
**Parent/carer trial**	**(*N* = 72)**	**(*N* = 99)**	

Sex, female, *n* (%)	37 (51.4)	48 (48.5)	0.9 (0.5 to 1.6)

Age group, 5–12 years, *n* (%)	25 (34.7)	37 (37.4)	1.0 (0.9 to 1.1)

Education, degree, *n* (%)	27 (38.0)^a^	53 (54.6)^a^	**2.0 (1.1 to 3.7)**

Index of Multiple Deprivation, lowest quintile (most deprived), *n* (%)	8 (11.3)^a^	10 (10.1)	0.9 (0.3 to 2.4)

Baseline severity, mean (SD)	12.3 (5.2)	13.3 (5.1)	1.04 (0.98 to 1.10)

Baseline emollient use, mean (SD)	12.1 (6.7)	11.7 (6.8)	1.04 (0.99 to 1.09)

Baseline total treatment use^b^	13.6 (8.6)	14.5 (7.8)	1.01 (0.97 to 1.06)

PETS, *n* (%)			
Symptoms too severe	36 (50.0)	61 (62.9)^a^	1.7 (0.9 to 3.1)
Uncertainty how to carry out treatment	18 (25.0)	44 (44.9)^a^	**2.4 (1.3 to 4.8)**
Doubt treatment efficacy	31 (43.7)^a^	60 (60.6)	**2.0 (1.1 to 3.7)**
Practical problems	33 (46.5)^a^	59 (59.6)	1.7 (0.9 to 3.1)

**Young people trial**	**(*N* = 72)**	**(*N* = 96)**	

Sex, female, *n* (%)	53 (73.6)	72 (75.0)	1.1 (0.5 to 2.2)

Age group, 18–25 years, *n* (%)	47 (65.3)	63 (65.6)	1.0 (0.5 to 1.9)

Index of Multiple Deprivation, lowest quintile (most deprivation), *n* (%)	7 (10.1)^a^	5 (5.3)^a^	0.5 (0.2 to 1.6)

Baseline POEM score, mean (SD)	13.8 (5.5)	16.1 (4.9)	**1.09 (1.02 to 1.16)**

Baseline emollient use score, mean (SD)	9.3 (6.4)	11.5 (7.2)	1.04 (0.99 to 1.10)

Baseline total treatment use score,^b^ mean (SD)	12.2 (7.3)	14.8 (8.5)	1.04 (0.99 to 1.08)

PETS, *n* (%)			
Symptoms too severe	46 (64.8)^a^	65 (69.2)^a^	1.2 (0.6 to 2.3)
Uncertainty how to carry out treatment	33 (45.8)	41 (43.6)^a^	0.9 (0.5 to 1.7)
Doubt treatment efficacy	54 (75.0)	74 (77.9)^a^	1.2 (0.6 to 2.4)
Practical problems	62 (87.3)^a^	80 (84.2)^a^	0.8 (0.3 to 1.9)

a *Denominator is different.*

b *Total treatment use is weekly combined emollient, topical corticosteroid, and topical calcineurin inhibitor use. PETS = Problematic Experiences of Therapy Scale. POEM = Patient-Oriented Eczema Measure. SD = standard deviation. Bold numbers indicate statistical significance.*

## Discussion

### Summary

Most participants in the intervention group met the minimum effective engagement threshold of viewing the core introductory content, suggesting a high level of user engagement. Users spent approximately 20 minutes on average on the interventions, demonstrating that positive outcomes on eczema severity depended on minimal time commitment from users. The study findings suggested that a substantial amount (30%–50%) of intervention effect on eczema severity at 24 weeks was mediated by increasing patient enablement. Among parents/carers, greater intervention engagement across 24 weeks was associated with higher levels of education, uncertainty about how to carry out treatment, and doubts about treatment efficacy at baseline. Among young people, higher intervention use was associated with higher baseline eczema severity.

Most of the associations between user characteristics and eczema severity were not statistically significant, indicating that there is little evidence to support a differential effect between user characteristics. However, this analysis was exploratory in nature, as the trial was not powered to detect differences between subgroups.

### Strengths and limitations

By including two different populations, this study was able to explore how implementation, mechanisms, and contextual factors may differ between groups. Trial participants received follow-up questionnaire email and/or SMS reminders prompting users to revisit the intervention. Time spent on the intervention was based on how long users spent on webpages, which may not be the same as actively engaging with the content. Therefore, the reported use is likely to be inflated.

Use of multiple timepoints allowed the exploration of changes in mediators and outcomes. However, in the RCTs, the primary outcome and potential mediators were measured at the same timepoint. Ideally, the mediators would be measured at an intermediate timepoint when the change is occurring, after the use of the intervention and before the measurement of the outcome at 24 weeks. However, the earliest measurement of the mediators after baseline was at 24 weeks when both mediator and outcome had changed significantly.

In the mediation analysis, total treatment use was included as a potential mediator. The effect for emollient use and topical corticosteroid/topical calcineurin inhibitor use were not explored separately, but there is unlikely to be a mediating effect as they were not statistically significant, and the effect sizes were small.

The study also had a high proportion of females in both groups and excluded those with very mild eczema, which may have limited the generalisability of the findings.

### Comparison with existing literature

The findings of the current study support previous research demonstrating that digital interventions can successfully enhance an individual’s ability to understand and cope with health issues (patient enablement).^26^^–^^28^ However, in both RCTs, there were no significant differences between groups in self-reported treatment use (the trials’ key hypothesised mechanisms) or perceived barriers to treatments. This finding may be a result of the challenges around measuring the complexities of treatment adherence in eczema. Only frequency of treatment use was measured, but participants in a qualitative process evaluation reported additional positive treatment outcomes, such as increasing the quantity (rather than frequency) of emollients used, starting topical corticosteroids more promptly after a flare-up, or reducing their use of topical corticosteroids to prevent overuse.^13^ Participants in the qualitative study also cited eczema-specific treatment barriers not captured by the PETS, including uncertainty about why treatments are used and the difference between the two treatments, and concerns about the long-term safety of treatments.

At baseline, higher eczema severity, higher level of education (among parents/carers), and having doubts and uncertainties about treatment use were significantly associated with higher intervention engagement. One explanation for these relationships is that the beliefs, concerns, and knowledge gaps may have motivated these participants to use the intervention. This is in line with the qualitative findings that suggested that participants who believed they had high levels of eczema knowledge, good eczema control, and a good treatment regimen tended to be less engaged with the intervention.^13^

### Implications for research and practice

This quantitative process evaluation suggests that the positive outcomes from the associated RCTs depended on only a minimal time commitment from users, providing further support that the wider implementation of Eczema Care Online (https://eczemacareonline.org.uk) is justified. Furthermore, the findings demonstrate that patient enablement is likely to mediate a substantial proportion of the effect of the intervention on eczema severity. However, other mechanisms, such as adherence to treatment regimens, management of irritants/triggers, and treatment concerns, are likely to also play a combined role. Future research should explore how these interventions are used and experienced by a more diverse cohort of people with eczema and their families in a real-world setting, outside a trial context. It would be useful to explore the extent to which users’ eczema-specific treatment concerns explain changes in intervention outcomes and associations between time since diagnosis and intervention outcome.

## Data

The data that support the findings of this study are available from the corresponding author on reasonable request.
